# Does the natural course of COVID‐19 infection include radiographic cystic changes?

**DOI:** 10.1002/ccr3.5036

**Published:** 2022-01-23

**Authors:** Noriko Hirai, Ryotaro Kida, Hiraku Yanada

**Affiliations:** ^1^ Respiratory Center Asahikawa Medical University Asahikawa Japan

**Keywords:** computed tomography, COVID‐19, cyst, pneumonia, pneumothorax

## Abstract

A patient with late phase of COVID‐19 pneumonia presented peripheral cystic features on chest computed tomography, which were spontaneously resolved with no antibiotic therapy or surgery. Physicians should pay attention to follow up the late phase of COVID‐19 pneumonia for better understanding its progression and clinical course.

## Graphical abstract

Clinicians should pay attention to the cystic features in the late phase of COVID‐19 pneumonia. Spontaneous resolution can occur, and observation alone with radiographic follow‐up may be appropriate for a subset of patients.

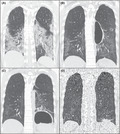

## 1 CLINICAL IMAGE

**FIGURE 1:**
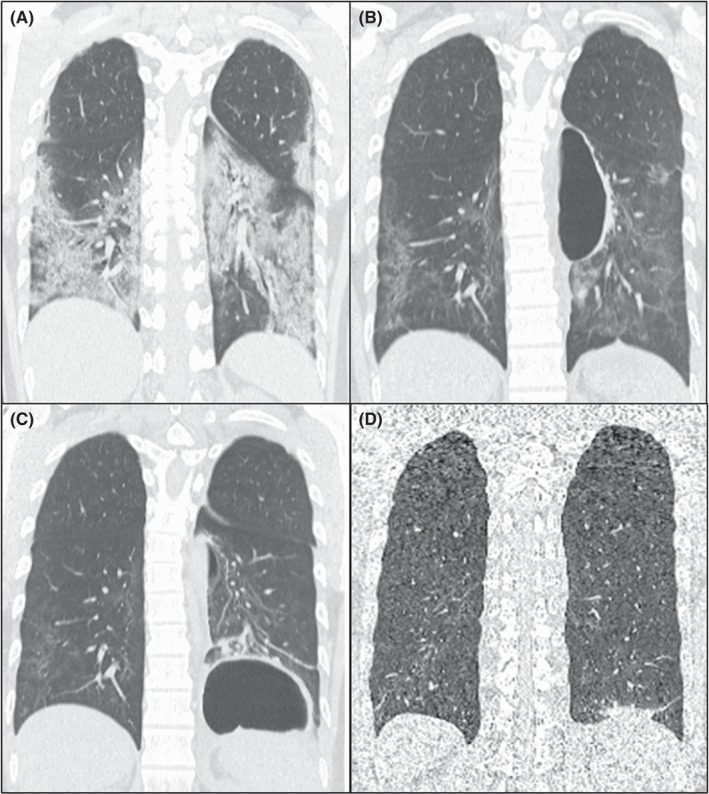
(A) Coronal computed tomography (CT) on admission demonstrated bilateral patchy consolidation and ground‐glass opacities (GGO). (B) Coronal CT on discharge revealed a cystic feature in the left mediastinal side. Bilateral consolidation and GGO were attenuated. (C) Coronal CT after four weeks from discharge demonstrated an additional cystic feature in the left epiphrenic side. (D) Coronal CT after eight weeks from discharge revealed disappearance of the cysts, bilateral consolidation, and GGO

A 35‐year‐old man with fever, cough, and worsening shortness of breath was hospitalized with COVID‐19 pneumonia. The patient’s chest computed tomography (CT) demonstrated bilateral patchy consolidation and ground‐glass opacities (GGO) (Figure 1A). The patient received oxygen and ciclesonide inhalation. Mechanical ventilation was not required. Twenty‐six days after, CT on discharge revealed a cystic feature in the left mediastinal side (Figure 1B). As the patient was physically recovered and the blood test did not show any inflammatory findings, only CT follow‐up was conducted. Four weeks after discharge, a new cystic feature enclosed by thickened wall was found in the epiphrenic side (Figure 1C). Although secondary bacterial or fungal infection was radiological differential diagnoses, again being asymptomatic without any blood test abnormalities prompted us to continue only radiological follow‐up. Eight weeks after discharge, both cysts had spontaneously disappeared without intervention (Figure 1D).

COVID‐19 pneumonia is a viral infection which is reported to present with GGO, patchy shadowing, and interstitial abnormalities.^1^ Cystic features and pleural involvement are less reported except for mechanical ventilation, but becoming recognized as one of a spontaneous complication.^1^, ^2^ Its progression is still poorly understood, but it may be caused by parenchymal ischemic damage and fibrosis.^2^ Physicians should pay attention to the late phase of COVID‐19 infection and its clinical course. Some physicians would consider referring such patients for thoracic surgery consultation. We want to emphasize spontaneous resolution of the cystic features can occur, and observation alone with radiographic follow‐up may be appropriate for a subset of patients.

## CONFLICTS OF INTEREST

The author declares that there is no conflict of interest that could be perceived as prejudicing the impartiality of the research reported.

## AUTHOR CONTRIBUTIONS

NH designed the conception and acquisition of the data, and all authors wrote the manuscript.

## ETHICAL APPROVAL

Not required.

## CONSENT

The patient provided written informed consent for his data to be published.

## Data Availability

Data sharing is not applicable to this article as no new data were created or analyzed in this study.
